# 肺大泡相关性肺癌2例并文献复习

**DOI:** 10.3779/j.issn.1009-3419.2011.09.13

**Published:** 2011-09-20

**Authors:** 亦鸣 虞, 敏 是, 琳 陈, 亮 陈, 晓东 赵, 在春 邓

**Affiliations:** 1 315020 宁波，宁波大学医学院附属医院呼吸科 Department of Respiratory Medicine, Affiliated Hospital of Medical College of Ningbo University, Ningbo 315020, China; 2 315200 宁波，浙江省宁波市龙赛医院呼吸科 Department of Respiratory Medicine, Longsai Hospital, Ningbo 315200, China; 3 315010 宁波，浙江省宁波市第二医院呼吸科 Department of Respiratory Medicine, Ningbo No.2 Hospital, Ningbo 315010, China; 4 315010 宁波，浙江省宁波市第一医院呼吸科 Department of Respiratory Medicine, Ningbo No.1 Hospital, Ningbo 315010, China; 5 315020 宁波，宁波大学医学院附属医学院胸外科 Department of General Thoracic Surgery, Affiliated Hospital of Medical College of Ningbo University, Ningbo 315020, China

**Keywords:** 肺肿瘤, 肺大泡, 诊断, Lung neoplasms, Bullae, Diagnosis

## Abstract

**背景与目的:**

肺癌多表现为肺部实质性占位，而以肺大泡为影像学表现的肺癌较为少见。本文通过报道2例肺大泡相关性肺癌并复习相关文献，旨在提高对肺大泡相关性肺癌的认识。

**方法:**

本文将报道2例肺大泡相关性肺癌患者的临床表现、影像资料及诊治过程，并结合相关文献报道进行分析。

**结果:**

肺大泡相关性肺癌是原发性肺癌的一种特殊形式，多发生于吸烟男性，临床表现隐匿，早期较难确诊，且易被漏诊、误诊。

**结论:**

肺大泡患者是肺癌的高危人群，应密切进行影像随访或进一步检查，对不能排除肺癌者应积极手术治疗。

## Abstract (en)

**Background and objective:**

Most of lung cancers present with a parenchymal mass. Lung cancers that present as bullae under imaging are unusual. The aim of the current article is to raise awareness regarding bullae-associated lung cancer through two case reports and their corresponding review of literature.

**Methods:**

The clinical, auxiliary examination data, and the diagnosis of two patients with bullae-associated lung cancer are presented and the relevant literature are reviewed.

**Results:**

Most cases of bullae-associated lung cancer are male heavy smokers. Considering the incidence of pulmonary carcinoma associated with bullous disease is high, radiographic findings would aid in the early detection of a malignant lesion.

**Conclusion:**

Bullous lung disease is a risk factor for lung cancer, and male, middle-age patients with bullae who smoke should be followed up by chest computed tomography and further examination. If the bullae may be diagnosed as lung cancer, an exploratory thoracotomy should be performed as early as possible.

肺癌是最常见的肺原发性恶性肿瘤，绝大多数肺癌起源于支气管粘膜上皮，故亦称支气管肺癌。近50多年来，世界各国特别是工业发达国家，肺癌的发病率和死亡率均迅速上升。肺癌多表现为肺部实质性占位，而以肺大泡为影像学表现的肺癌较为少见，现报道2例肺大泡相关性肺癌，并复习相关文献，以提高对此类肺癌的认识。

## 1 临床资料

病例1：患者男性，80岁，因“反复痰血1年”入院。患者1年前因受凉出现咳嗽、咳痰，痰中带少量血丝，无发热及盗汗，当地医院胸部CT（图 1A）检查示“左上肺大泡形成，左肺上叶少许纤维化病灶”，支气管镜检查无异常，血生化全套、肿瘤全套、血沉、PPD试验等均无异常，经抗感染治疗后痰血减少而出院。8个月前，因痰血反复就诊，胸部CT（图 1B）示“左肺尖陈旧性肺结核伴空洞形成”，复查支气管镜无异常，考虑“结核性支气管扩张伴感染”，予抗感染治疗后痰血减少。6个月前复查胸部CT（图 1C）示“左上肺结核性空洞伴曲菌球形成”，予诊断性抗结核治疗1个月后，胸部CT（图 1D）示“病灶略有缩小”，但因肝功能损害而停止抗结核治疗。2个月前胸部CT（图 1E）示“左上肺病灶较前增大”，为进一步诊治而入院。既往体健，曾吸烟1包/天×30年，已戒10年。查体：神清，浅表淋巴结无肿大，气管居中，胸廓无畸形，双肺语颤对称，叩诊清音，呼吸音清，未闻及干湿性啰音，心腹查体正常。入院后三大常规、PPD、血沉、生化全套、肿瘤全套等均无异常。为明确诊断而行经电视胸腔镜下探查，术中发现：左上肺肺尖部与胸壁粘连严重，肿块位于尖部，4 cm×3.5 cm，内后壁局部侵犯胸膜；术后常规病理报告：中分化鳞癌，送检淋巴结阴性；送检肺组织中未见结核性病变。

**1:**
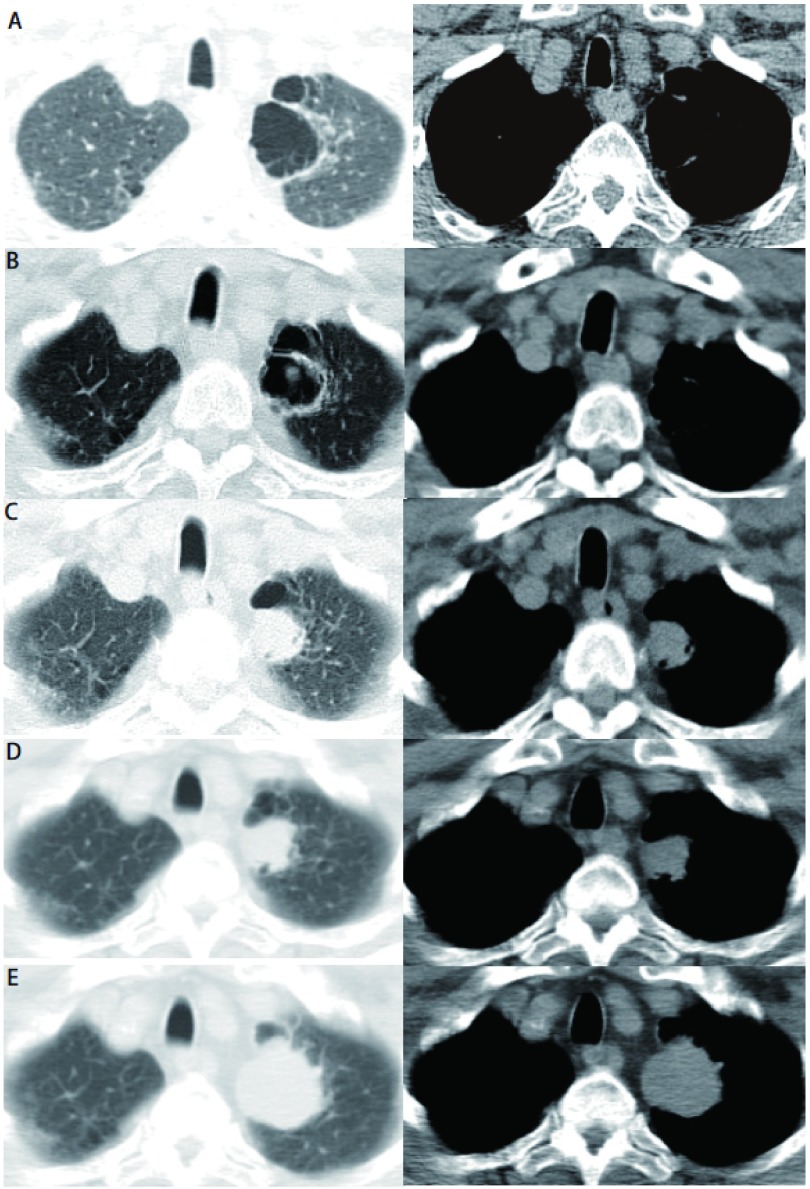
图A-E为病例1病灶由肺大泡到肺部肿块的演变过程。A：CT见左上肺大泡；B：2个月后肺大泡内出现结节；C-E：6个月内结节逐渐增大成为肿块。 Pictures A-E show the lesion experienced a succesion of variations from bullae to mass of case one. A: CT scan shows bullae in the upper lobe of left lung; B: A nodule can be found in the bullae two months later; C-E: The nodule becomes a mass gradually in six months.

病例2：患者男性，40岁，因“咳嗽伴左胸痛1个月”入院。患者1个月前出现无明显诱因干咳，伴左侧持续性胸痛，咳嗽时加剧，无畏寒、发热，无咯血，无乏力、盗汗，当地医院胸片（图 2A）示“右上肺空洞，肺结核？”，胸部CT（图 2B）示“右肺上叶前段可见空洞影，壁薄，局部见局限性增厚，外缘与侧胸壁胸膜有粘连表现，纵隔可见肿大淋巴结”，考虑为“肺结核”至结核病专科医院住院治疗。住院期间痰找抗酸杆菌5次均阴性，血结核抗体阴性，PPD（+），两次检测血癌胚抗原（carcino-embryonic antigen, CEA）分别为289.20 ng/mL、329.10 ng/mL，行胸部CT增强扫描示“右肺上叶空洞内结节影，增强动脉期、静脉期未见明显强化”。因患者肺结核诊断依据不足，为进一步诊治转至本院。既往体健，无烟酒嗜好。入院查体：浅表淋巴结无肿大，气管居中，胸廓无畸形，胸壁无压痛，双肺触觉语颤对称，叩诊清音，呼吸音清，无干湿性啰音，心腹查体正常。患者入院后三大常规、凝血功能、血生化等检查均见异常，痰找抗酸杆菌2次阴性，血CEA为335.4 ng/mL。上腹部CT平扫未见明显异常，胃镜检查提示“慢性浅表性胃炎”。PET-CT检查报告为“右肺上叶肺癌伴双侧肺门、锁骨上淋巴结、左侧肋骨等多发转移”。为获得病理诊断而行电视胸腔镜下右肺上叶楔形切除术。术中见肿瘤位于右肺上叶前段，约2 cm，未与壁层胸膜粘连，边界欠清，肿瘤中央空洞坏死，呈灰白色。术后病理报告为“右上肺鳞腺混合癌”，术后予化疗。

**2:**
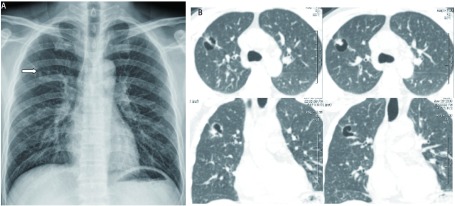
图A、B分别为病例2的胸片及胸部CT表现。A：胸片见右肺上叶一空腔性病变；B：胸部CT见右上肺含壁结节的空腔，空腔周围可见毛刺及胸膜凹陷征。 Pictures A and B are the shows of radiograph and CT scan of case two. A: Chest X-ray shows a cavity in the right upper lobe; B: CT shows a cavity with mural nodule in the right upper lobe. Radioactive burr and pleural indentation sign can be found around the cavity.

## 2 讨论

本文报道了2例发生于肺大泡内壁的肺癌。因肺大泡位于肺上叶，易被误诊为“肺结核空洞”、“结核性空洞伴曲菌球形成”，但患者病程中均无结核中毒症状。影像学上，2例患者均表现为肺大泡内壁小结节，病例1则在胸部CT随访中发现肺大泡内结节性病变逐渐进展，最后占据整个肺大泡。

历史上，Bass等^[1]^于1951年首次报道发生于肺大泡的肺癌。肺大泡相关性肺癌的名称最早由Tsutsui等^[2]^在1988年提出，它是指发生于肺大泡内、肺大泡壁上或邻近肺大泡的原发性肺癌。据文献^[3-5]^报道，肺大泡相关性肺癌的发生率为3.5%-4.2%，并认为肺大泡患者患肺癌的风险较无肺大泡患者高32倍^[6]^，组织学分型发现鳞癌的发生率明显高于腺癌，低分化和未分化癌占一半以上。肺大泡相关性肺癌的发生，究其原因可能与肺大泡好发于吸烟者有关，而吸烟是肺癌和肺大泡的共同高危因素；其次，对非吸烟者而言，肺大泡壁由压缩的肺组织与结缔组织组成，肺大泡与周围气道的气流相对受限，病原菌及环境中的有害物质易沉积于肺大泡壁，导致肺大泡壁的反复慢性炎症和纤维疤痕组织形成，这个过程可导致肺大泡周围致癌物的积累，并最终导致疤痕癌的形成^[5]^。肺癌可发生于肺大泡，但肺癌本身也可导致肺大泡的形成。在肺癌的发生过程中，致癌物可抑制抗弹性蛋白酶的活性，从而导致肺泡间隔的破坏和肺大泡的形成；同时，癌组织可造成支气管的狭窄和闭塞，引起气流受限而形成肺大泡^[5]^。Tsutsui等^[2]^总结了肺大泡相关性肺癌的影像学特点：（1）结节影位于或邻近肺大泡；（2）大泡壁部分或弥漫性增厚；（3）出现大泡的继发表现，如大泡直径的改变、液体的潴留及气胸。因此在临床工作中，必须对肺大泡内壁欠光整或内壁存在小结节的患者保持高度的重视，严密随访，及早发现恶性肿瘤。

Hirai等^[7]^回顾了日本1980年-2005年报道的34例肺大泡相关性肺癌的临床特点：肺大泡相关肺癌的平均发病年龄为（52.7±9.9）岁；所有患者均为男性，其中绝大多数有吸烟史；所有患者中病灶发生于肺上叶者的30例（89%），仅2例发生于双肺上叶，1例发生于右肺下叶，这与肺大泡的好发部位相符；从确诊手段上分析，18例（53%）患者为手术病理，11例为经支气管镜活检，2例为经皮肺活检，1例为纵隔镜，另有2例确诊手段不明确。从上述数据可以看出，通过非创伤性手段如痰找脱落细胞学检查确诊非常困难，而由于气管镜下活检及经皮肺活检在肺大泡患者中易出现气胸、血气胸，且因取得的组织较小，易出现假阴性结果，因此手术组织病理在该病中诊断率最高。

肺大泡相关性肺癌缺少特异性表现，多数患者为在常规胸部影像学检查时偶然发现，近60%的肺大泡相关性肺癌患者首诊时并未发现肺癌，而在随访中明确诊断^[8]^，其中1例患者在诊断肺大泡10年之后被诊断为肺癌^[9]^。由此可见，肺大泡患者定期进行胸部影像学随访对于及早发现肺大泡相关性肺癌非常重要。

作为肺癌的一种特殊形式，肺大泡相关性肺癌在早期较难确诊，且易被漏诊、误诊。临床医生应该充分认识到：肺大泡患者属于肺癌高危人群，对肺大泡患者应进行影像学随访或进一步检查，对不能排除肺癌者应积极手术治疗。
